# No association between XRCC1 gene Arg194Trp polymorphism and risk of lung cancer: evidence based on an updated cumulative meta-analysis

**DOI:** 10.1007/s13277-014-1745-z

**Published:** 2014-03-04

**Authors:** Jing Zhang, Xian-Tao Zeng, Jun-Rong Lei, Yi-Jun Tang, Jiong Yang

**Affiliations:** 1 Department of Respiratory Medicine, Zhongnan Hospital, Wuhan University, 169 Donghu Road, Wuhan, 430071 China; 2 Department of Respiratory Medicine, Taihe Hospital, Hubei University of Medicine, Shiyan, 442000 China; 3 Center for Evidence-Based Medicine and Clinical Research, Taihe Hospital, Hubei University of Medicine, Shiyan, 442000 China; 4 Department of Neurosurgery, Taihe Hospital, Hubei University of Medicine, Shiyan, 442000 China

**Keywords:** X-ray repair cross-complementing group 1, XRCC1, Lung cancer, Polymorphism, Risk, Meta-analysis

## Abstract

X-ray repair cross-complementing group 1 (XRCC1) gene Arg194Trp polymorphism has been reported to be associated with risk of lung cancer in many published studies. Nevertheless, the research results were inconclusive and conflicting. To reach conclusive results, several meta-analysis studies were conducted by combining results from literature reports through pooling analysis. However, these previous meta-analysis studies were still not consistent. Hence, we used an updated and cumulative meta-analysis to get a more comprehensive and precise result from 25 case–control studies searching through the PubMed database up to September 1, 2013. The meta-analysis was carried out by the Comprehensive Meta-Analysis software and the odds ratio (OR) with 95 % confidence interval (CI) was used to estimate the pooled effect. The result involving 8,876 lung cancer patients and 11,210 controls revealed that XRCC1 Arg194Trp polymorphism was not associated with lung cancer risk [(OR = 0.97, 95 %CI = 0.92–1.03) for Trp vs. Arg; (OR = 0.92, 95 % CI = 0.85–0.98) for ArgTrp vs. ArgArg; (OR = 1.07, 95 % CI = 0.92–1.23) for TrpTrp vs. ArgArg; (OR = 0.93, 95 % CI = 0.87–1.00) for (TrpTrp + ArgTrp) vs. ArgArg; and (OR = 1.08, 95 % CI = 0.94–1.25) for TrpTrp vs. (ArgTrp + ArgArg)]. The cumulative meta-analysis showed that the results maintained the same, while the ORs with 95 % CI were more stable with the accumulation of case–control studies. The sensitivity and subgroups analyses showed that the results were robust and not affected by any single study with no publication bias. Relevant studies might not be needed for supporting these results.

## Introduction

X-ray repair cross-complementing group 1 (XRCC1) is involved in base excision repair protein that located on chromosome 19q13.2–13.3 with a length of 33 kb [1–4]. The polymorphisms of XRCC1 gene have been identified as three categories of codons 194(Arg to Trp), 280(Arg to His), and 399 (Arg toGln) [5, 6]. One of them, Arg194Trp polymorphism was first reported in 1998 by Shen and coworkers [7]. In 2001, David-Beabesand coworkers [8] found that Arg194Trp polymorphism might contribute to lung cancer in African-American and Caucasian. Ratnasinghe and coworker [5] found similar results in Chinese during the same year. Later on, many molecular epidemiological studies reported the association of XRCC1 Arg194Trp with lung cancer susceptibility [5, 8–30]. However, these results remain conflicting and inconclusive. To reach conclusive results, several meta-analysis studies were conducted by combining results across studies from literatures through pooling analysis. However, these previous meta-analysis investigations were still not consistent [31–33]. Furthermore, new published research studies were coming out, but the inconclusive results are still a problem to be resolved. Therefore, the association of Arg194Trp with lung cancer susceptibility with lung cancer risk remains unclear.

In order to obtain more comprehensive and precise results, we conducted cumulative meta-analysis [34, 35] to explore the truly association between Arg194Trp polymorphism and lung cancer risk based on 25 case–control studies. The meta-analysis is reported based on preferred reporting items for systematic reviews and meta-analyses (PRISMA) [36] statement.

## Material and methods

### Inclusion criteria

A study met all of the following inclusion criteria was included: (1) to evaluate the association between XRCC1 Arg194Trp polymorphism and risk of lung cancer; (2) cohort or case–control design and the patients were diagnosed by histology or pathology; (3) the number of genotype distribution in both case and control group were directly reported or calculated from the reported data; and (4) the published language was English or Chinese.

### Search strategy

The search terms [(“XRCC1” or “X-ray repair cross-complementing group 1”) and “polymorphism” and (“lung cancer” or “lung carcinoma”)] were used to search the PubMed database up to September 1, 2013. The reference list of the included articles and relevant meta-analyses were manually searched.

### Data extraction

**Fig. 1:**
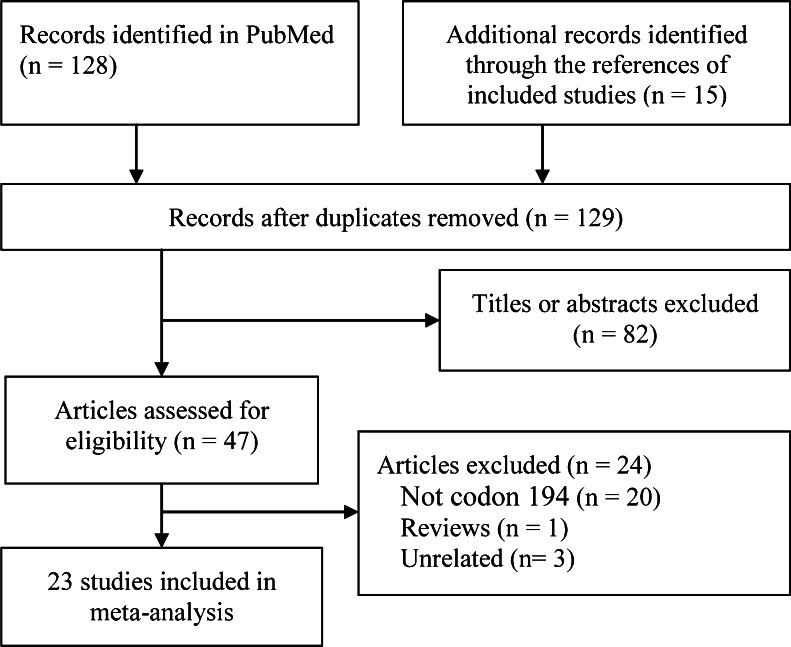
Flow chart from identification of eligible studies to final inclusion

Two authors independently chose 25 case–control studies, which were illustrated in Fig. 1. The data were independently extracted by authors according to the pre-specified table. The following data were extracted: the surname of first author, publication year, country origin and ethnicity, study design, cancer type, source of control, number and genotyping distribution of cases and controls, genotyping method, Hardy–Weinberg equilibrium (HWE) for controls. Disagreements were resolved through discussion with the third author.

### Statistical analysis

Five genetic models [Trp vs. Arg; ArgTrp vs. ArgArg; TrpTrp vs. ArgArg; (TrpTrp + ArgTrp) vs. ArgArg; and TrpTrp vs. (ArgTrp + ArgArg)] were used to calculate the pooled odds ratio (OR) and its 95 % confidence interval (CI) to present the strength of associations between XRCC1 Arg194Trp polymorphism and risk of lung cancer. The fixed-effects model was used firstly, if heterogeneity among included studies was detected by *I* ^2^ statistics (*I* ^2^ ≤ 40 %) [37], we shifted to random-effects model. Subgroups analysis were conducted based on the ethnicity, source of controls, cancer types, study design, and HWE for controls.

The influence of sample size on the overall risk estimation was carried out by cumulative meta-analysis [35], and the influence of single study on the overall risk estimation was determined through sensitivity analysis by omitting one study each time. The publication bias was detected by funnel plot analysis. All the analysis was performed using the Comprehensive Meta-Analysis software, version 2.2 (Biostat, Englewood, New Jersey) [38].

## Results

### Study section and characteristics

The electronic searching yielded 128 studies, and the hand searching yielded 15 studies initially; finally, 23 articles involving 25 case–control studies [5, 8–29] contained 8,876 lung cancer patients and 11,210 controls were included. Figure 1 presents flow chart of study selection. The main characteristics of these eight studies were shown in Table 1. Of them, three were multicenter studies [12, 16, 17], two articles were included two case–control studies [8, 24], and only one study was out of HWE [27].

**Table 1:** Characteristics of included studies

References	Country (ethnicity)	Case				Source of control	Control				Genotyping	HWE
		*N*	ArgArg	ArgTrp	TrpTrp		*N*	ArgArg	ArgTrp	TrpTrp		
David-Beabes [8]	USA (Caucasian)	180	158	22	0	PB	461	407	54	0	PCR-RFLP	0.39
David-Beabes [8]	USA (African-Americans)	154	142	10	2	PB	243	205	36	2	PCR-RFLP	0.67
Ratnasinghe [5]	China (Asian)	108	52	47	9	PB	216	85	104	21	TaqMan	0.22
Chen [9]	China (Asian)	109	48	44	11	PB	109	57	40	5	PCR-RFLP	0.79
Chan [10]	China (Asian)	75	50	22	3	HB	162	79	67	16	PCR-RFLP	0.71
Hu [11]	China (Asian)	710	335	311	64	HB	710	339	308	63	PCR	0.59
Hung [12]	European (Caucasian)	2,188	1,878	259	10	HB	2,198	1,828	292	12	PCR	0.87
Schneider [13]	Germany (Caucasian)	446	389	53	4	HB	622	544	75	3	PCR	0.74
Shen [14]	China (Asian)	118	65	41	12	HB	112	64	40	8	PCR	0.62
Hao [15]	China (Asian)	1,024	524	409	91	PB	1,118	572	459	87	PCR	0.77
Landi [16]	Europe (Caucasian)	295	118	143	34	HB	314	123	149	42	PCR	0.96
Matullo [17]	Europe (Caucasian)	116	98	16	2	PB	1,094	951	141	2	TaqMan	0.22
Zienolddiny [18]	Norway (Caucasian)	336	309	26	1	PB	405	368	35	2	TaqMan	0.23
De Ruyck [19]	Belgium (Caucasian)	110	101	8	1	HB	110	93	17	0	PCR-RFLP	0.38
Pachouri [20]	India (Asian)	103	40	39	24	PB	122	52	47	23	PCR-RFLP	0.051
Yin [21]	China (Asian)	241	120	98	23	HB	249	119	109	21	PCR-RFLP	0.65
Li [23]	China (Asian)	350	184	136	30	HB	350	196	133	21	PCR-RFLP	0.89
Improta [22]	Italy (Caucasian)	94	42	41	11	HB	121	53	61	7	PCR-RFLP	0.15
Chang [24]	USA (Latinos)	113	89	23	1	PB	299	223	66	10	Illumina	0.1
Chang [24]	USA (African–Americans)	255	221	34	0	PB	280	248	31	1	Illumina	0.97
Tanaka [25]	Japan (Asian)	50	28	15	7	PB	50	25	23	2	PCR	0.47
Janik [26]	Poland (Caucasian)	88	64	24	0	HB	79	51	28	0	PCR-SSCP	0.55
Buch [27]	USA (Caucasian)	720	682	36	2	HB	928	839	83	6	Illumina	0.03
Wang [28]	China (Asian)	209	105	83	21	HB	256	137	96	23	PCR-RFLP	0.59
Guo [29]	China (Asian)	684	314	302	68	HB	602	265	274	63	PCR-LDR	0.58

*N* total sample size, *PB* population-based controls, *HB* hospital-based controls, *HWE* Hardy–Weinberg equilibrium, *PCR*-*RFLP* polymerase chain reaction-restriction fragment length polymorphism, *PCR*-*LDR* polymerase chain reaction-ligase detection reaction, *PCR*-*SSCP* polymerase chain reaction-single strand conformation polymorphism

### Meta-analysis

**Fig. 2:**
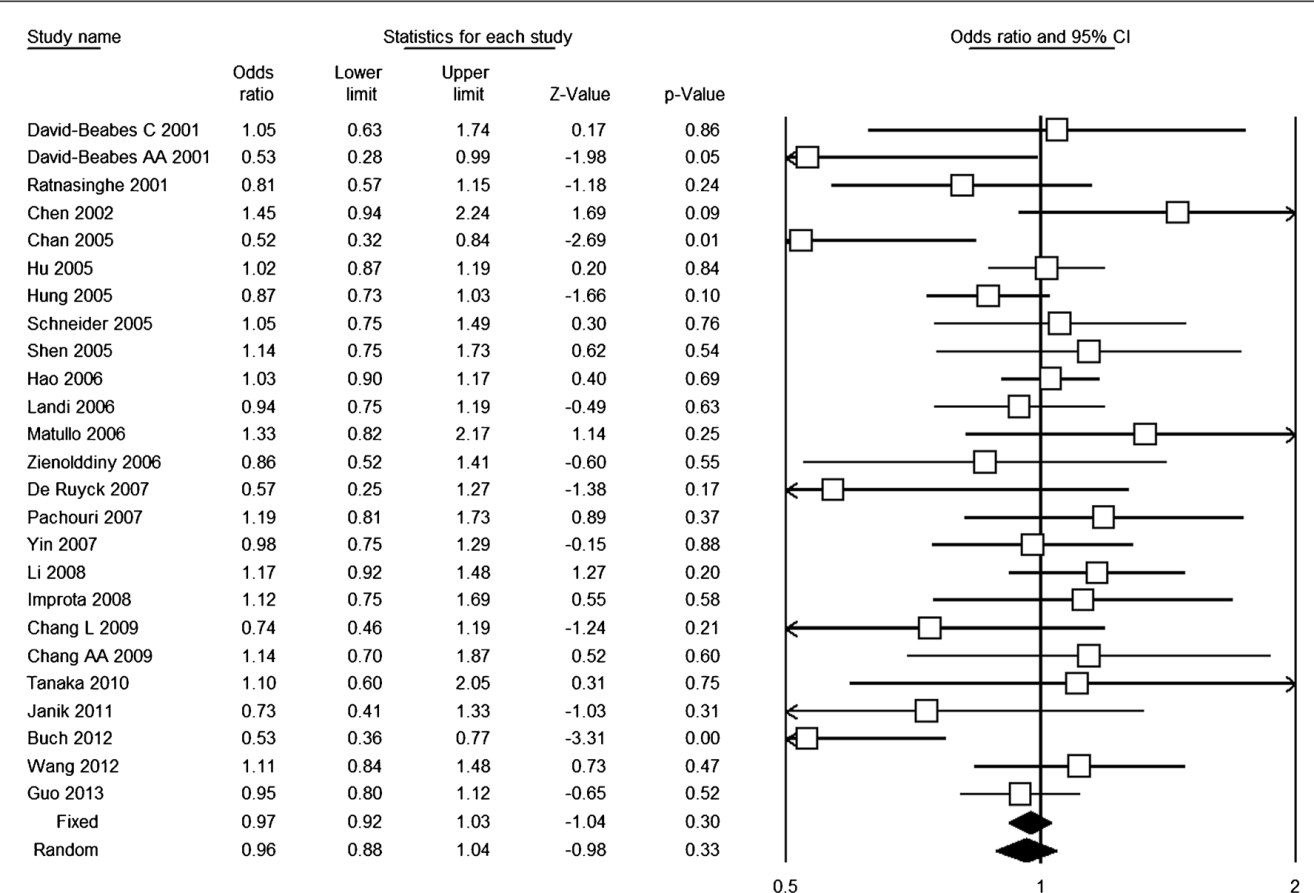
Forest plot based on Trp vs. Arg genetic model

Table 2 presented the overall and subgroups results of XRCC1 Arg194Trp polymorphism and lung cancer risk. Overall, the heterogeneity of all five genetic models were acceptable (*I* ^2^ ≤ 40 %), and meta-analysis based on fixed-effects model showed that there was no association of XRCC1 Arg194Trp polymorphism with risk of lung cancer [(OR = 0.97, 95 % CI = 0.92–1.03) for Trp vs. Arg, Fig. 2; (OR = 0.92, 95 % CI = 0.85–0.98) for ArgTrp vs. ArgArg; (OR = 1.07, 95 % CI = 0.92–1.23) for TrpTrp vs. ArgArg; (OR = 0.93, 95 % CI = 0.87–1.00) for (TrpTrp + ArgTrp) vs. ArgArg; and (OR = 1.08, 95 % CI = 0.94–1.25) for TrpTrp vs. (ArgTrp + ArgArg)].

**Table 2:** Results of overall and subgroup meta-analysis

	No. of studies	Trp vs. Arg			ArgTrp vs. ArgArg			TrpTrp vs. ArgArg			TrpTrp + ArgTrp vs. ArgArg			TrpTrp vs. ArgTrp + ArgArg		
		OR (95 % CI)	*p* for OR	*I* ^2^ (%)	OR (95 % CI)	*p* for OR	*I* ^2^ (%)	OR (95 % CI)	*p* for OR	*I* ^2^ (%)	OR (95 % CI)	*p* for OR	*I* ^2^ (%)	OR (95 % CI)	*p* for OR	*I* ^2^ (%)
Total	25	0.97 (0.92–1.03)	0.30	38.8	0.92 (0.85–0.98)	0.017	17.4	1.07 (0.92–1.23)	0.380	9.5	0.93 (0.87–1.00)	0.047	28.5	1.08 (0.94–1.25)	0.255	3.8
Ethnicity																
Asian	12	1.02 (0.95–1.09)	0.662	28.3	0.97 (0.88–1.06)	0.479	0.0	1.10 (0.93–1.29)	0.258	7.7	0.99 (0.91–1.08)	0.825	13.7	1.11 (0.95–1.30)	0.717	0.0
Caucasian	10	0.89 (0.80–0.98)	0.024	37.6	0.85 (0.76–0.96)	0.008	16.1	1.01 (0.70–1.44)	0.974	29.0	0.86 (0.76–0.97)	0.011	24.7	1.02 (0.72–1.44)	0.914	32.6
Others	3	0.80 (0.59–1.07)	0.130	46.4	0.83 (0.60–1.16)	0.276	66.8	0.50 (0.15–1.67)	0.259	0.0	0.81 (0.59–1.11)	0.189	59.1	0.52 (0.15–1.74)	0.287	0.0
Source of controls																
HB	14	0.95 (0.88–1.01)	0.113	48.7	0.90 (0.83–0.99)	0.022	28.2	1.02 (0.85–1.22)	0.847	0.0	0.91 (0.84–0.99)	0.035	40.0	1.03 (0.87–1.23)	0.725	0.0
PB	11	1.01 (0.92–1.12)	0.816	20.9	0.94 (0.83–1.07)	0.375	5.6	1.17 (0.91–1.50)	0.217	26.6	0.97 (0.86–1.10)	0.657	10.6	1.20 (0.94–1.52)	0.144	23.2
HWE																
Yes	24	0.97 (0.91–1.02)	0.530	20.8	0.93 (0.87–1.00)	0.067	0.0	1.08 (0.93–1.25)	0.317	8.3	0.95 (0.89–1.02)	0.163	8.0	1.09 (0.95–1.26)	0.211	2.5
No	1	0.53 (0.36–0.77)	0.001	–	0.53 (0.36–0.80)	0.002	–	0.41 (0.08–2.04)	0.276	–	0.53 (0.35–0.78)	0.001	–	0.43 (0.09–2.13)	0.300	–

*OR* odds ratio, *CI* confidence interval, *PB* population-based controls, *HB* hospital-based controls, *HWE* Hardy–Weinberg equilibrium

**Fig. 3:**
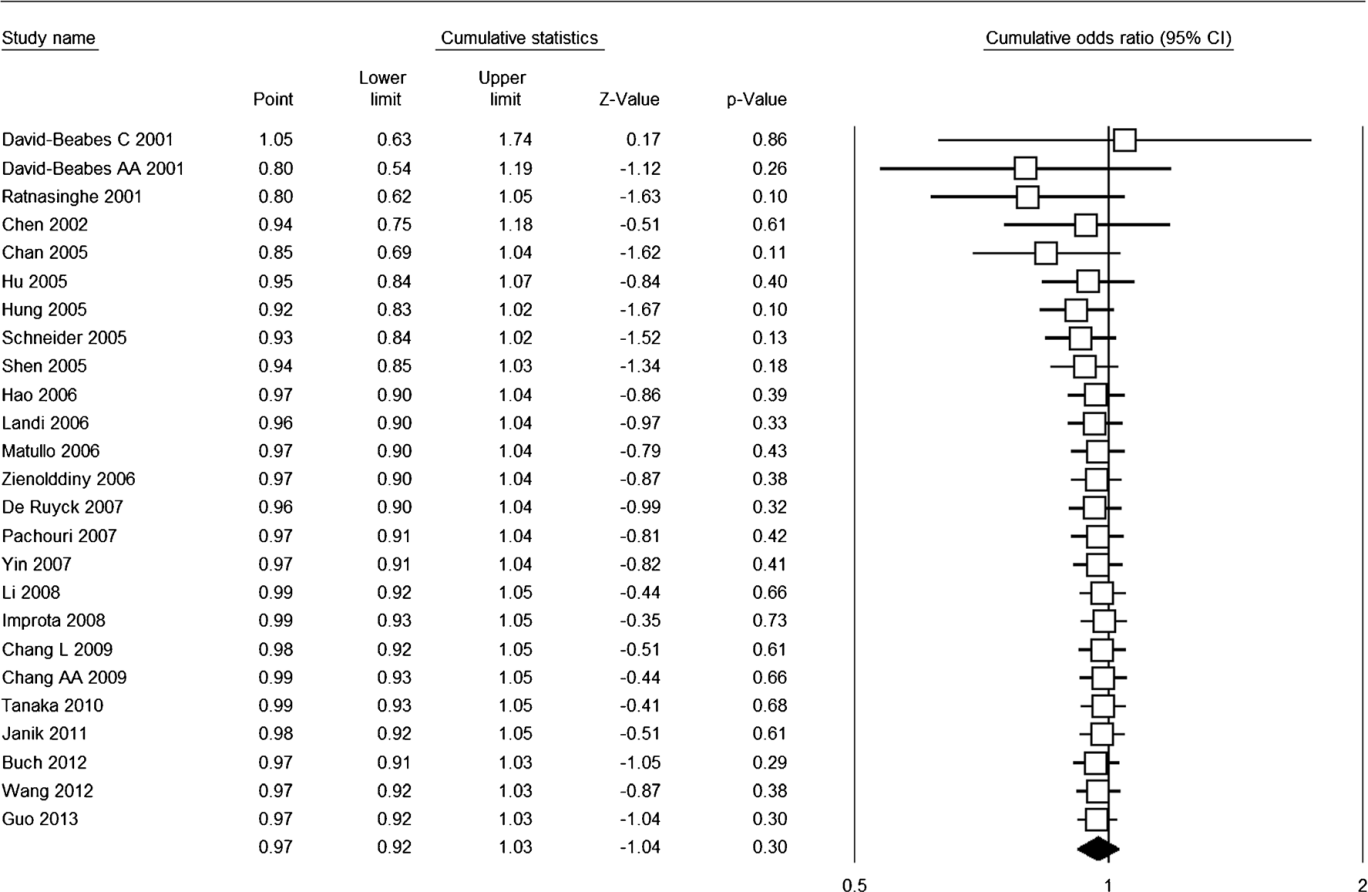
Forest plot for cumulative meta-analysis based on Trp vs. Arg genetic model

**Fig. 4:**
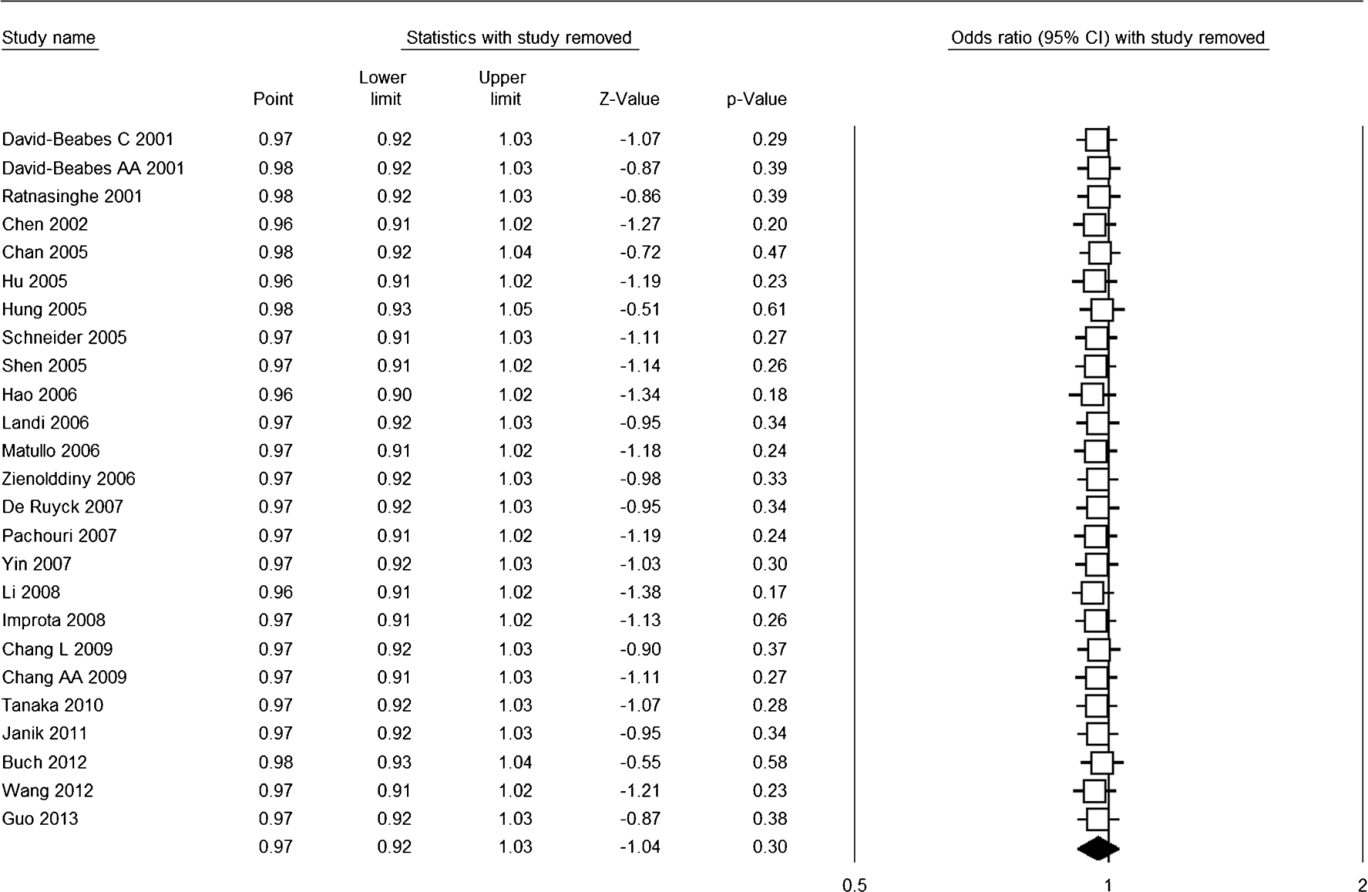
Forest plot for sensitivity analysis based on Trp vs. Arg genetic model

The cumulative meta-analysis accumulated the studies according to the publication year and showed that there was no significant association between XRCC1 Arg194Trp polymorphism and lung cancer risk (Fig. 3). The sensitivity analysis showed that the results were robust and were not influenced by any single study (Fig. 4), with ORs in the range of 0.96–0.98 and 95 % CIs in the range of 0.90–1.05. Subgroup analysis upon source of control, ethnicity, and HWE also revealed similar results (Table 2).

### Publication bias

**Fig. 5:**
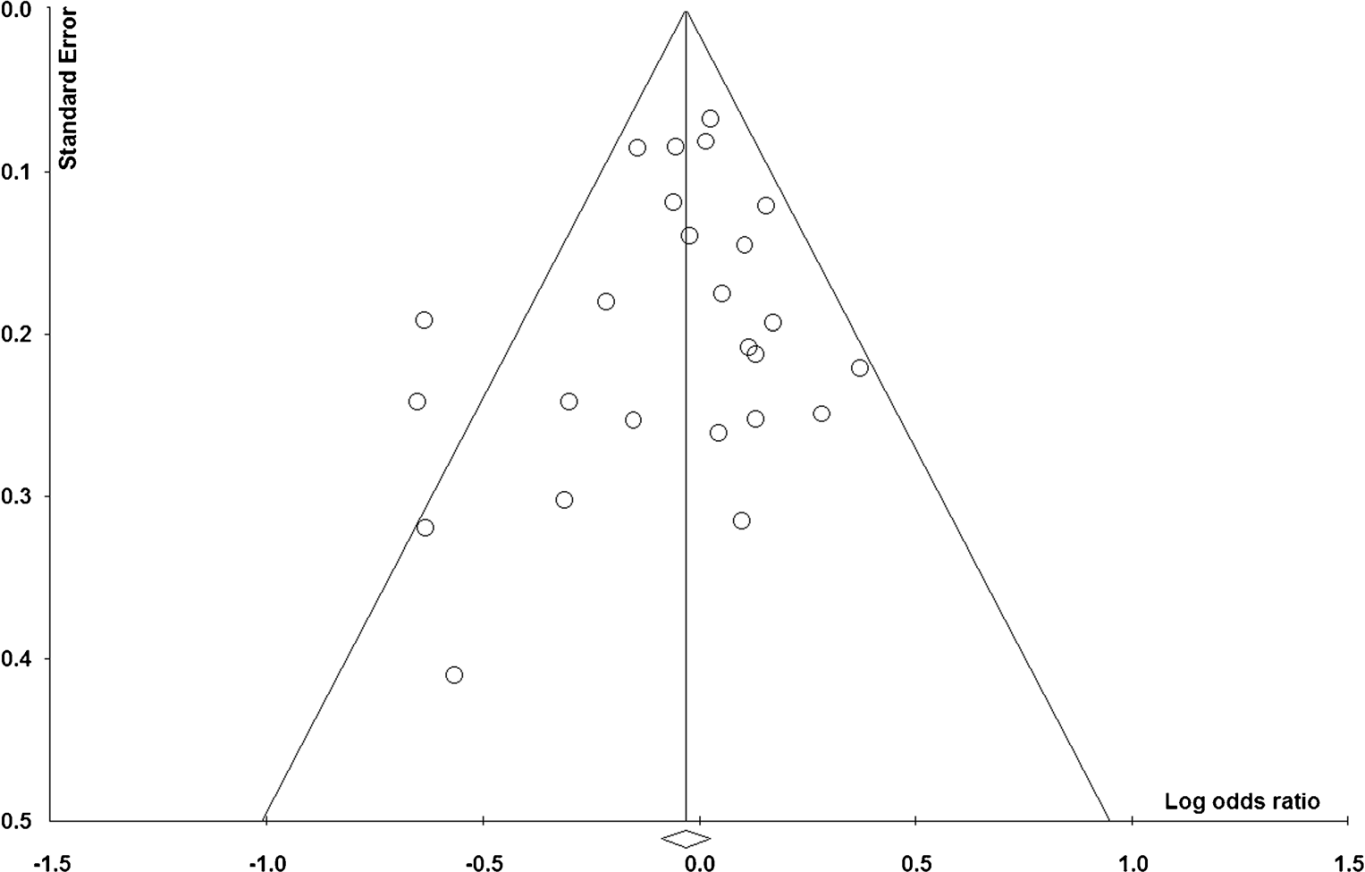
Funnel plot based on Trp vs. Arg genetic model

Figure 5 shows the funnel plot of based on Trp vs. Arg genetic model. The relatively symmetric distribution indicated that there was no publication bias, which was confirmed by Egger’s test [(*p* = 0.33 for Trp vs. Arg; *p* = 0.12 for ArgTrp vs. ArgArg; *p* = 0.65 for TrpTrp vs. ArgArg; *p* = 0.25 for (TrpTrp + ArgTrp) vs. ArgArg; and *p* = 0.50 for TrpTrp vs. (ArgTrp + ArgArg))].

## Discussion

Meta-analysis is a statistical method of combining results across studies from literatures to resolve discrepancy in genetic association studies [39]. The meta-analysis of 25 case–control studies indicated that XRCC1 Arg194Trp polymorphism is not associated with lung cancer risk within human populations, and subgroup analysis upon source of controls, ethnicity, and HWE for controls is consistent with this result, which was also supported by cumulative meta-analysis and sensitivity analysis.

Compared to previously meta-analyses [31–33], the included studies of our analysis are most precise and comprehensive attributing to the largest sample size and accumulative meta-analysis method. Hence, the results are more precise and comprehensive. In addition, cumulative meta-analysis was performed to investigate the tendency of results by accumulating single study year by year. This analysis could be used to determine whether new relevant studies are needed or not. Indeed, we found that the results remained the same when studies were accumulated. Coincidentally, the sensitivity analysis indicated that the results were not influenced by any single study. Hence, our results were more precise and useful for appropriate care in lung cancer.

Obviously, there were potential to moderate level heterogeneity. From the subgroups analysis, we found that ethnicity and source of control might not be the source of heterogeneity (Table 2). When we deleted the study reported by Buch et al.[27], which was not according to HWE any more, the heterogeneity of all genetic models were decreased and the results of all five genetic models were of no significance (Table 2). This further indicated that violations and deviations in HWE might be one source of heterogeneity and do largely influence the results [40].

There were some limitations of our meta-analysis. First, there was heterogeneity among included studies. Although the heterogeneity was probably from the study reported by Buch et al. [27], we could not conclude whether the heterogeneity came from ethnicity or inconsistent results. Obviously, the homogeneity of Asians and Caucasian was good, but only the one combined with mixed ethnicities was significant. Second, although no obvious publication bias was detected; the funnel plot was not very symmetry. Our meta-analysis is limited to language and database restrictions. The PubMed database is the only search source and included published studies were either in English or Chinese [28]. Third, this meta-analysis was based on unadjusted data, lacking of detailed genotype information stratified by main confounding variables from original studies. Therefore, gene-gene and gene-environment interactions remain unclear.

In conclusion, this meta-analysis suggests that XRCC1 Arg194Trp polymorphism is not associated with lung cancer risk, either in Asians or Caucasians, either the controls were sourced with or without HWE. These results were not influenced by any single study, and relevant studies are not needed for supporting this result. Due to the limitations of this meta-analysis, current results should be viewed with caution and future studies should be conducted in gene-gene and gene-environment interactions.
